# Supplementary material for: Lipidomic and in-gel analysis of maleic acid co-polymer nanodiscs reveals differences in composition of solubilized membranes
Source: Commun Biol. 2021 Feb 16;4:218. doi: 10.1038/s42003-021-01711-3 (PMC7886889; doi:10.1038/s42003-021-01711-3)
Supplement: Supplementary file 1 — Supplementary Information [file 42003_2021_1711_MOESM1_ESM.pdf]

Electronic Supporting Information

Lipidomic and in-gel analysis of maleic acid co-polymer nanodiscs reveals differences in composition of solubilized membranes

Marta Barniol-Xicota[a] and Steven H. L. Verhelst * [a,b]

[a] KU Leuven – University of Leuven, Laboratory of Chemical Biology, Department of Cellular and Molecular Medicine, Herestraat 49 box 802, 3000 Leuven, Belgium

[b] Leibniz Institute for Analytical Sciences ISAS, AG Chemical Proteomics, Otto-Hahn-Str. 6b, 44227 Dortmund, Germany

Index

Supplementary Method	S1
Supplementary Figure S1	S4
Supplementary Figure S2	S5
Supplementary Figure S3	S6
Supplementary Figure S4	S6
Supplementary Figure S5	S7
Supplementary Figure S6	S8
Supplementary Figure S7	S9
Supplementary Figure S8	S10
Supplementary Figure S9	S11
Supplementary note	S12

Supplementary Method

IR-spectra of the xMA polymers

Styrene maleic acid (2.3:1)

IR (ATR) ν: 2927, 1566, 1492, 1411, 759, 698 cm⁻¹.

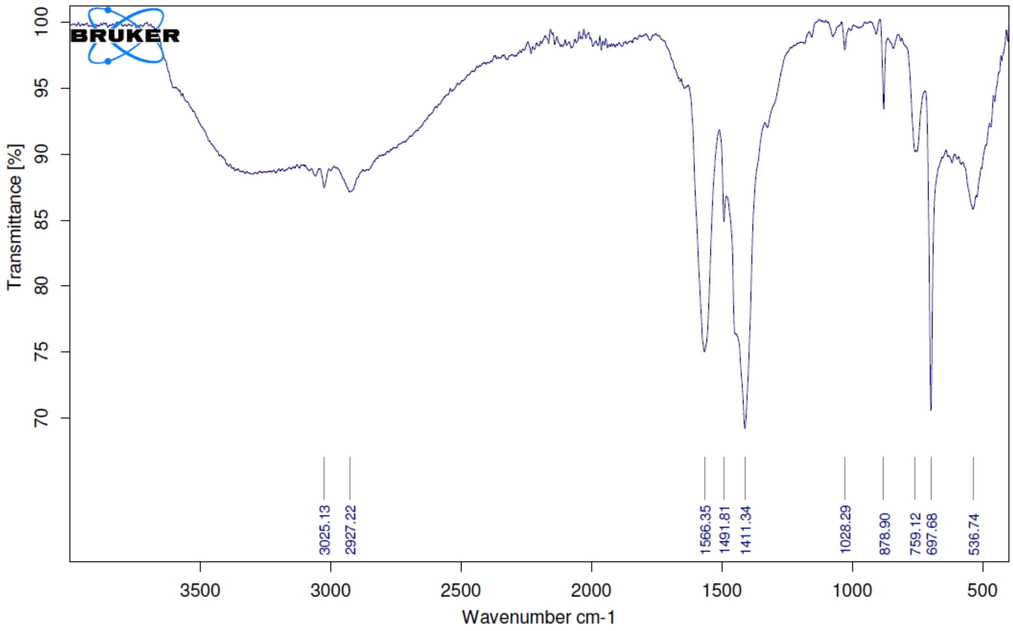

Diisobutylene maleic acid (1:1)

IR (ATR) ν: 2947, 1709, 1366, 1186, 921 cm⁻¹.

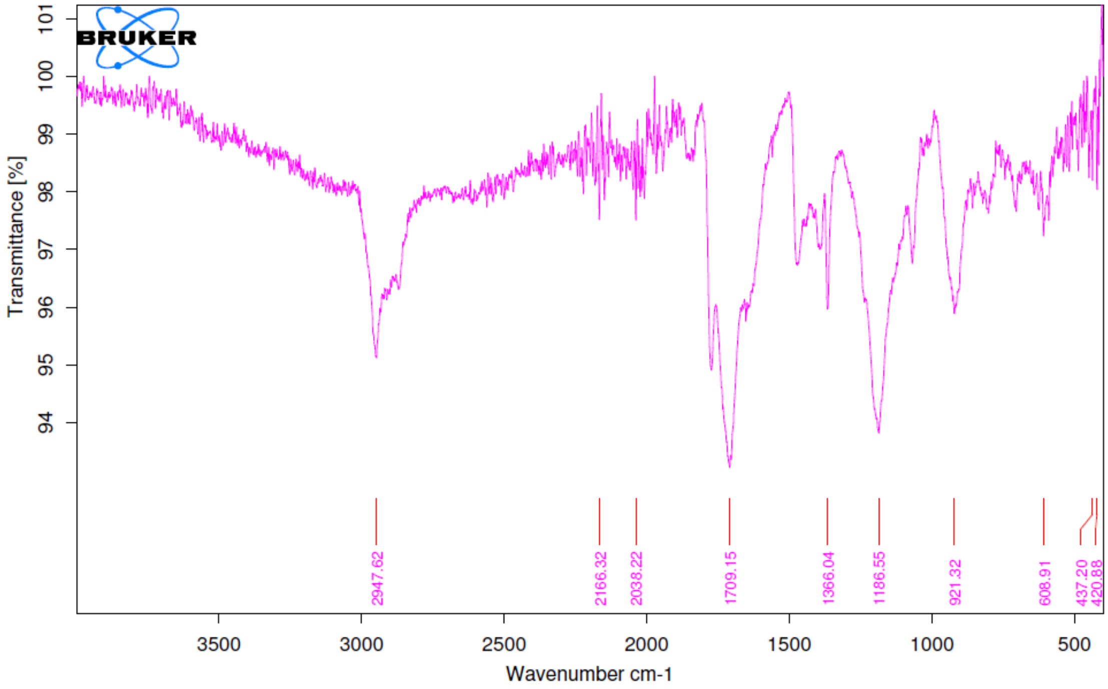

Styrene maleic anhydride (2.3:1)

IR (ATR) ν: 2923, 1855, 1774, 1493, 1453, 914, 698 cm⁻¹.

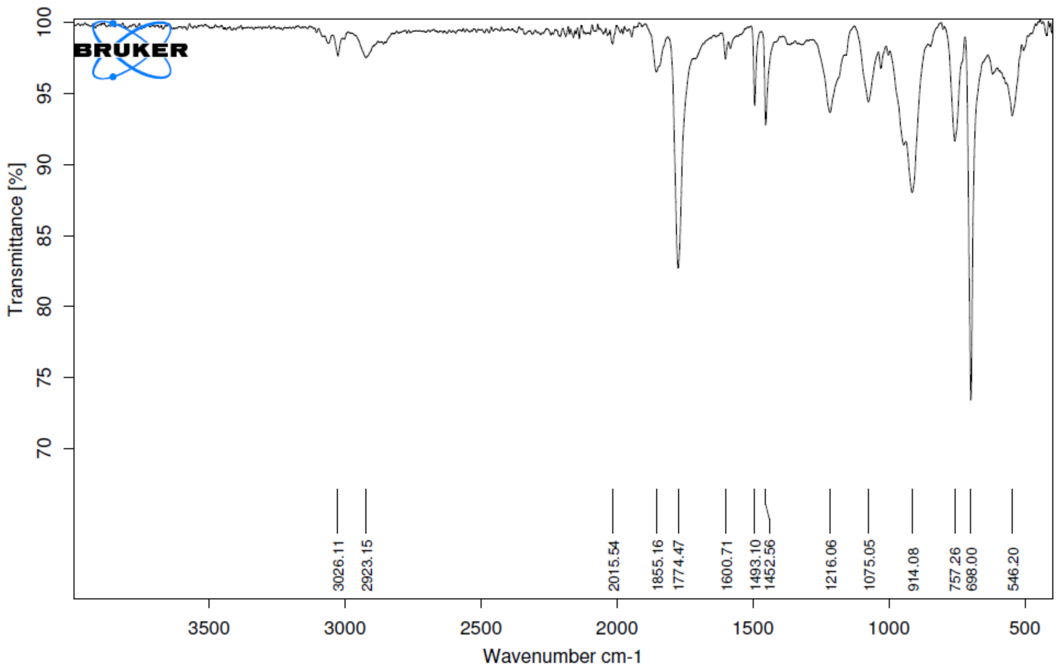

Styrene trimethyl-ethylamime maleimide (SMA-QA)

IR (ATR) ν: 3014, 1695, 1486-1146, 701 cm⁻¹.

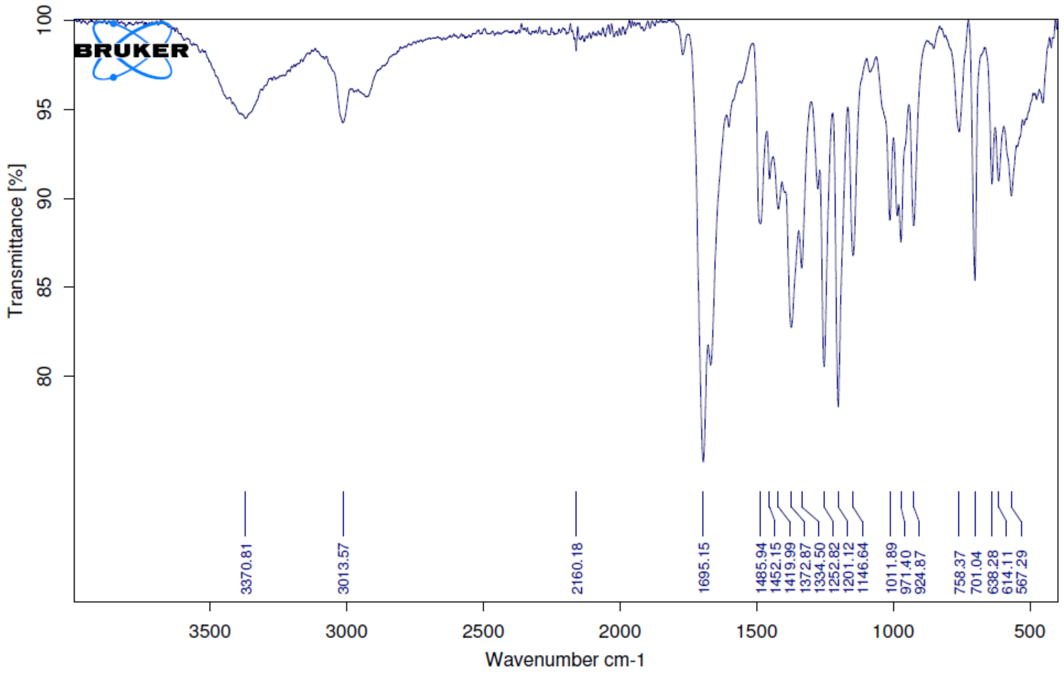

SMA QA vs SMAnh

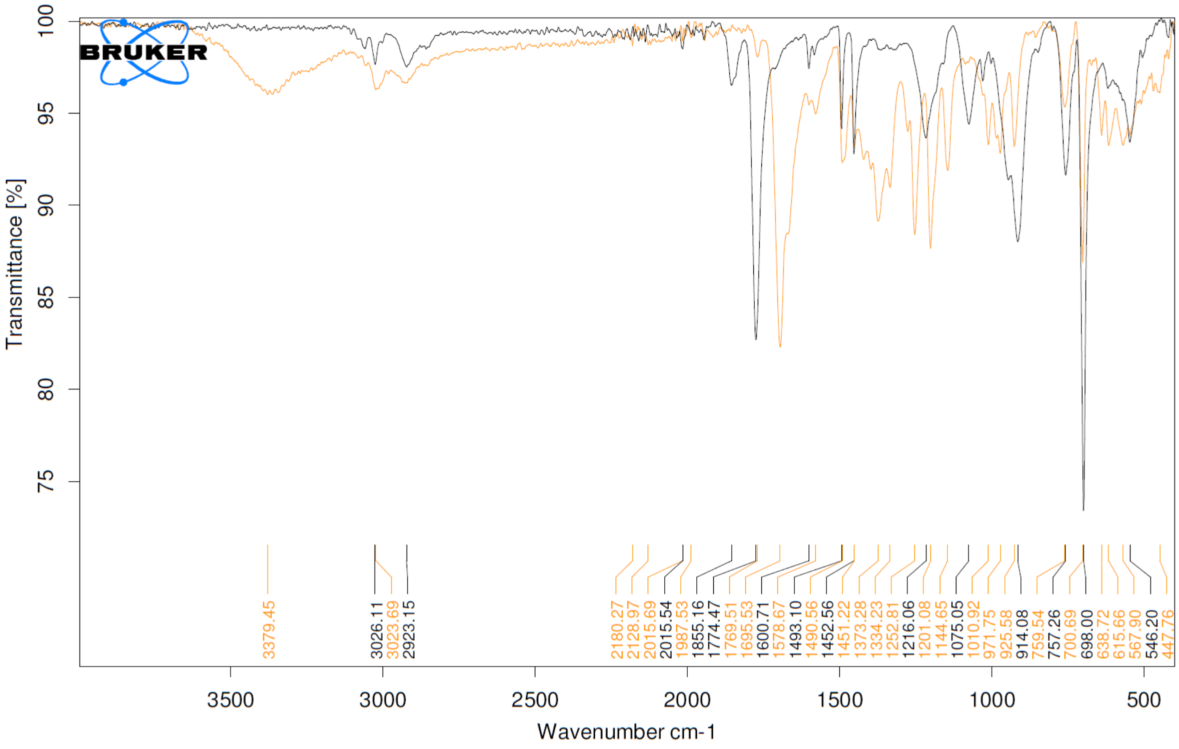

Superimposition of SMA-QA (in orange) and SMAnh IR (in grey) spectra.

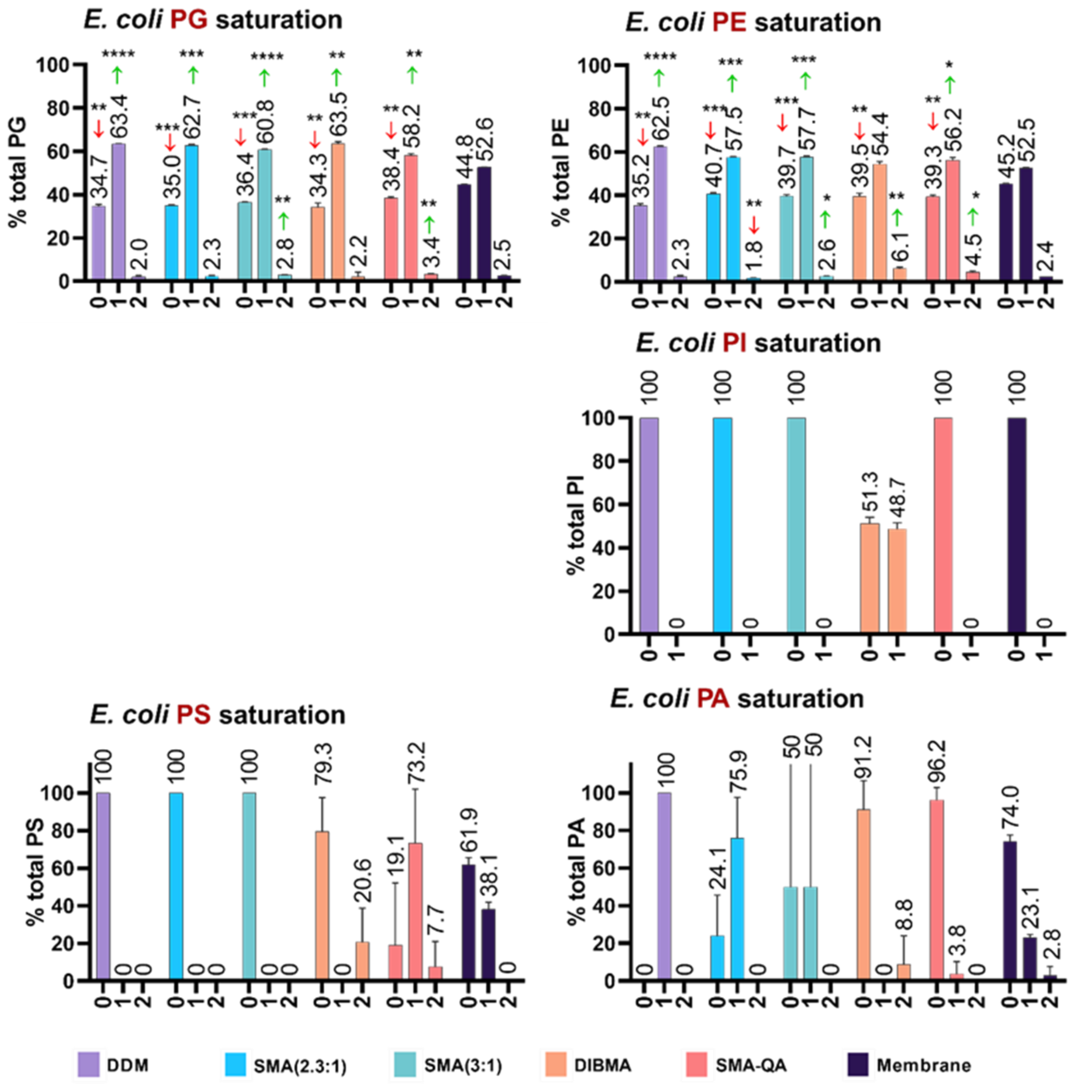

Figure S1. Saturation of PL species present in *E. coli* classified depending on their headgroup, being: PG, PE, PI, PS or PA . The number of double bonds indicated are those found in the total fatty acid chains of a single PL species‡.

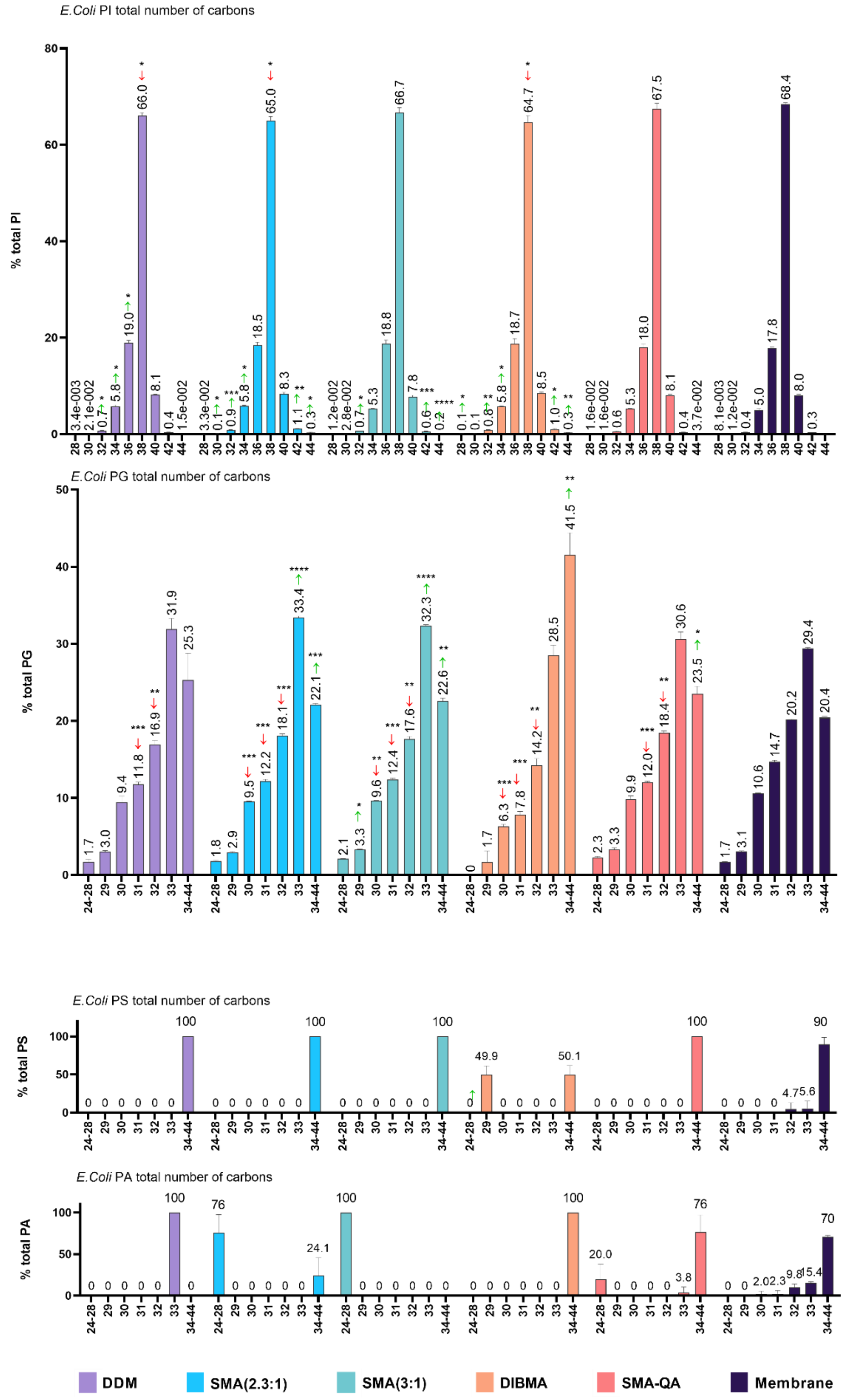

Figure S2. Fatty acid chain length distribution in *E. coli* samples represented by the number of carbons present in the fatty acid chains of the total PI, PG, PS or PA species‡.

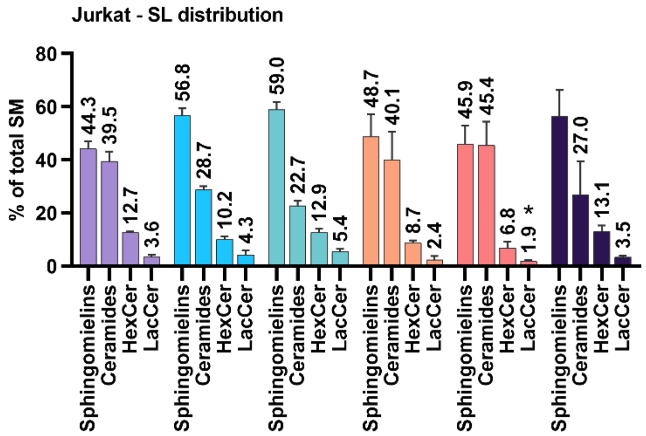

Figure S3. Distribution of species present in the SL fraction of solubilized Jurkat membranes‡.

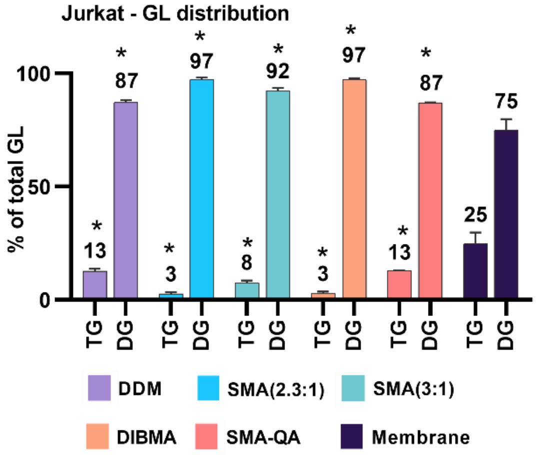

Figure S4. Distribution of species present in the GL fraction of solubilized Jurkat membranes‡. DG stands for diacylglycerol and TG for triacylglycerol‡.

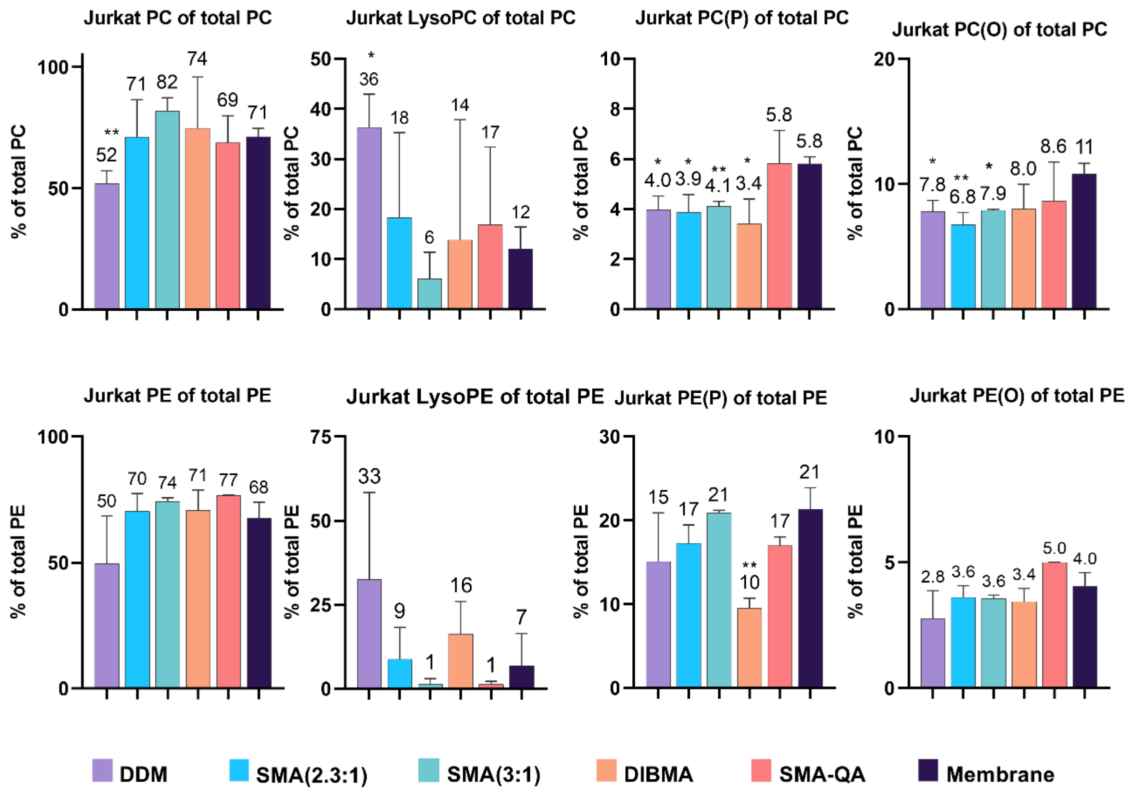

Figure S5. Analysis of the PC and PE fractions of Jurkat membranes. The found different species are: intact PE or PC, being PE or PC with two acyl chains; lysoPE or lysoPC if one of the acyl chain is lost; PE(P) or PC(P) if one of the esther bonds is replaced by an alkenyl ether linkage and PE(O) or PC(O) when the esther linkage is replaced by an alkyl ether bond ‡.

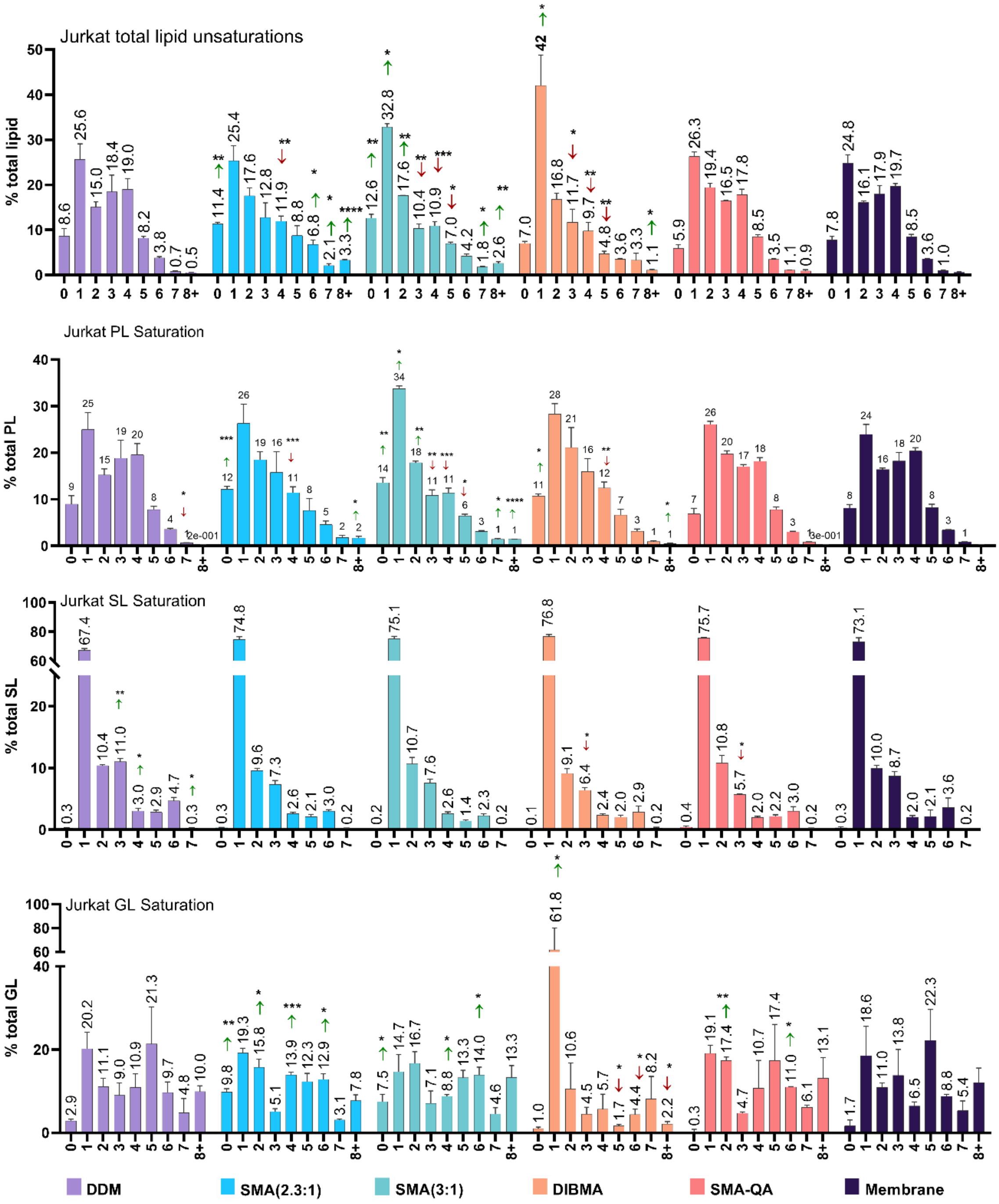

Figure S6. Saturation degree of Jurkat membranes. Represented from top to bottom, saturation profile of: all lipid species; PL; SL and GL ‡.

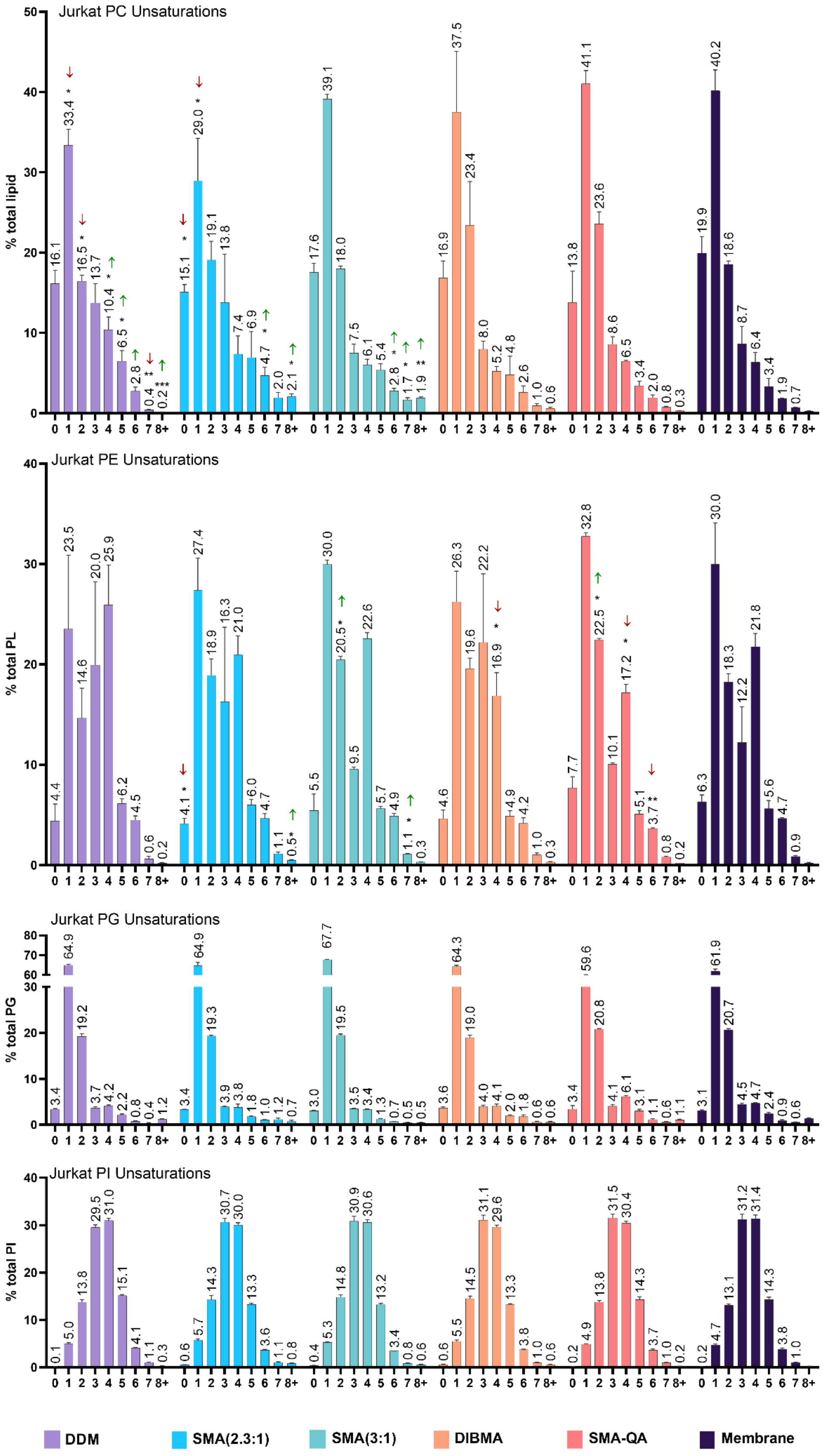

Figure S7. Saturation of PL species present in Jurkat membranes classified depending on their headgroup, being: PC, PG, PE or PI. The number of double bonds indicated are those found in the total fatty acid chains of a single PL species‡.

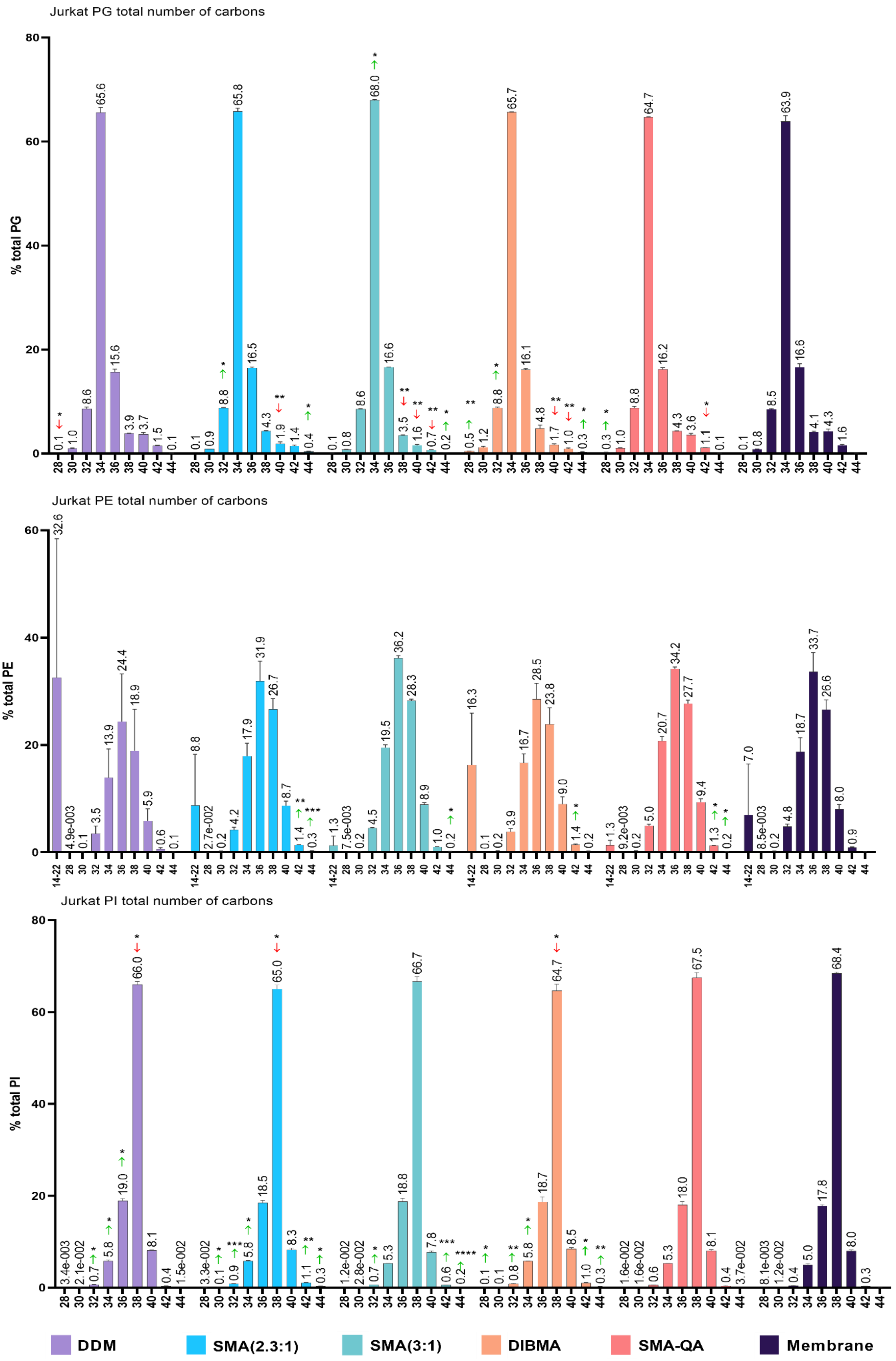

Figure S8. PL fatty acid chain length distribution in Jurkat samples represented by the number of carbons present in the fatty acid chains of the total PG, PE or PI species‡.

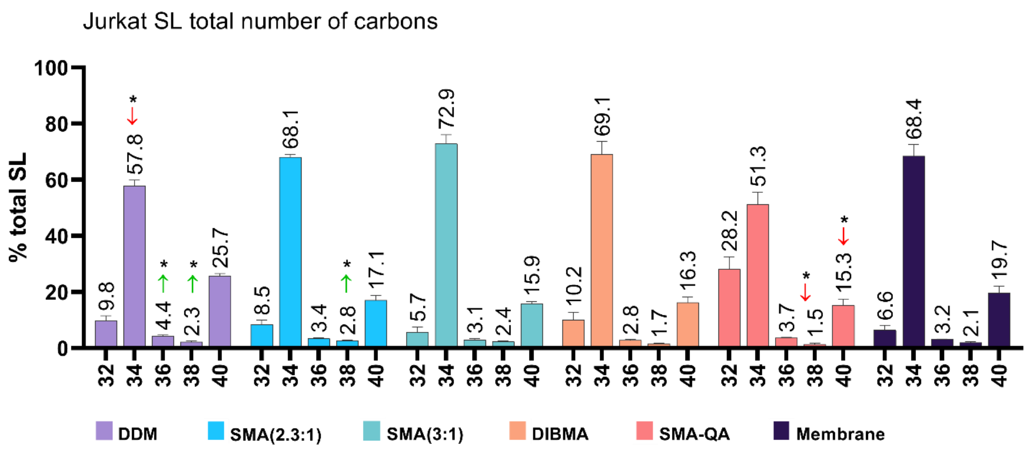

Figure S9. Fatty acid chain length distribution of the SL fraction present in Jurkat samples represented by the number of carbons present in the fatty acid chains‡.

Notes

‡ Data points correspond to three technical replicates. Error bars represent ± S.D. Significant differences (upon one-way ANOVA) are denoted as *($p < 0.05$), **($p < 0.01$), ***($p < 0.001$), ****($p < 0.001$).
