# Supplementary material for: Lipidomic and in-gel analysis of maleic acid co-polymer nanodiscs reveals differences in composition of solubilized membranes
Source: Commun Biol. 2021 Feb 16;4:218. doi: 10.1038/s42003-021-01711-3 (PMC7886889; doi:10.1038/s42003-021-01711-3)
Supplement: Supplementary file 3 — Description of Additional Supplementary Files [file 42003_2021_1711_MOESM3_ESM.pdf]

Description of Additional Supplementary Files

File name: Supplementary Data 1

Description: E. Coli lipidomics analysis data.

File name: Supplementary Data 2

Description: Jurkat Cells lipidomics analysis data.

File name: Supplementary Data 3

Description: Jurkat Cells cholesterol quantification data.
