# Supplementary material for: Lipidomic and in-gel analysis of maleic acid co-polymer nanodiscs reveals differences in composition of solubilized membranes
Source: Commun Biol. 2021 Feb 16;4:218. doi: 10.1038/s42003-021-01711-3 (PMC7886889; doi:10.1038/s42003-021-01711-3)
Supplement: Supplementary file 7 — Reporting Summary [file 42003_2021_1711_MOESM7_ESM.pdf]

nature research

Corresponding author(s): Steven H. L. Verhelst

Last updated by author(s): Jan 8, 2021

Reporting Summary

Nature Research wishes to improve the reproducibility of the work that we publish. This form provides structure for consistency and transparency in reporting. For further information on Nature Research policies, see our Editorial Policies and the Editorial Policy Checklist.

Statistics

For all statistical analyses, confirm that the following items are present in the figure legend, table legend, main text, or Methods section.

n/a	Confirmed	
☐	☒	The exact sample size (*n*) for each experimental group/condition, given as a discrete number and unit of measurement
☐	☒	A statement on whether measurements were taken from distinct samples or whether the same sample was measured repeatedly
☐	☒	The statistical test(s) used AND whether they are one- or two-sided<br>*Only common tests should be described solely by name; describe more complex techniques in the Methods section.*
☒	☐	A description of all covariates tested
☐	☒	A description of any assumptions or corrections, such as tests of normality and adjustment for multiple comparisons
☐	☒	A full description of the statistical parameters including central tendency (e.g. means) or other basic estimates (e.g. regression coefficient) AND variation (e.g. standard deviation) or associated estimates of uncertainty (e.g. confidence intervals)
☒	☐	For null hypothesis testing, the test statistic (e.g. F, t, r) with confidence intervals, effect sizes, degrees of freedom and P value noted<br>*Give P values as exact values whenever suitable.*
☒	☐	For Bayesian analysis, information on the choice of priors and Markov chain Monte Carlo settings
☒	☐	For hierarchical and complex designs, identification of the appropriate level for tests and full reporting of outcomes
☒	☐	Estimates of effect sizes (e.g. Cohen's d, Pearson's r), indicating how they were calculated

Our web collection on statistics for biologists contains articles on many of the points above.

Software and code

Policy information about availability of computer code

Data collection	Bruker Vertex 70 spectrometer spectrophotometer software; Molecular Devices ID3 platereader software
Data analysis	Prism 8 (GraphPad Software, USA); Microsoft Excel; MultiQuantTM software version 3.0.3.

For manuscripts utilizing custom algorithms or software that are central to the research but not yet described in published literature, software must be made available to editors and reviewers. We strongly encourage code deposition in a community repository (e.g. GitHub). See the Nature Research guidelines for submitting code & software for further information.

Data

Policy information about availability of data

All manuscripts must include a data availability statement. This statement should provide the following information, where applicable:

- Accession codes, unique identifiers, or web links for publicly available datasets
- A list of figures that have associated raw data
- A description of any restrictions on data availability

The data that support the findings of this study are available from the corresponding author upon reasonable request.

Field-specific reporting

Life sciences study design

All studies must disclose on these points even when the disclosure is negative.

Sample size	3 technical replicates for each sample, to ensure data significance.
Data exclusions	Once technical replicate data point was excluded for one specific sample (SMA-QA Jurkat). The reason for this decision being that the results from the lipidomics data collected were unfeasible taking into account the controls used and the fact that differed enormously from the other two replicates, within which the variation was minimal. This has been indicated in the manuscript.
Replication	Three technical replicates were used for the eukaryotic and procaryotic cell line analysis. In all cases the data was reproducible showing little variation between replicates. The solely exception for this is 1 technical replicate for SMA-QA in the Jurkat cell line, in that case only two technical replicates were used.
Randomization	n/a
Blinding	n/a

Reporting for specific materials, systems and methods

We require information from authors about some types of materials, experimental systems and methods used in many studies. Here, indicate whether each material, system or method listed is relevant to your study. If you are not sure if a list item applies to your research, read the appropriate section before selecting a response.

Materials & experimental systems

n/a	Involved in the study	
☐	☒	Antibodies
☐	☒	Eukaryotic cell lines
☒	☐	Palaeontology and archaeology
☒	☐	Animals and other organisms
☒	☐	Human research participants
☒	☐	Clinical data
☒	☐	Dual use research of concern

Methods

n/a	Involved in the study	
☒	☐	ChIP-seq
☒	☐	Flow cytometry
☒	☐	MRI-based neuroimaging

Antibodies

Antibodies used	Direct-Blot™ HRP anti-His Tag; BioLegend UK Ltd
Validation	-

Eukaryotic cell lines

Policy information about cell lines

Cell line source(s)	ATCC
Authentication	n/a
Mycoplasma contamination	All cell lines tested negative for mycoplasma contamination
Commonly misidentified lines (See ICLAC register)	-
